# *In vitro* characterization of representative clinical South African *Staphylococcus aureus* isolates from various clonal lineages

**DOI:** 10.1002/nmi2.53

**Published:** 2014-06-26

**Authors:** W F Oosthuysen, H Orth, C Lombard, B Sinha, E Wasserman

**Affiliations:** 1 Division of Medical Microbiology, Stellenbosch University, Tygerberg HospitalCape Town, South Africa; 2 National Health Laboratory Services, Microbiology Laboratory, Tygerberg Academic ComplexCape Town, South Africa; 3 Biostatistics Unit, Medical Research CouncilCape Town, South Africa; 4 University Medical Centre GroningenGroningen, The Netherlands; 5 Pathcare LaboratoriesCape Town, South Africa

**Keywords:** Cellular invasiveness, cytotoxic, methicillin-resistance, Panton–Valentine leucocidin, *Staphylococcus aureus*, South Africa

## Abstract

Data concerning the virulence and pathogenesis of South African strains of *Staphylococcus aureus* are limited. We investigated host–pathogen interactions of randomly selected clinical *S. aureus* isolates representing various clones. We characterized the ability of isolates to adhere to fibronectin, fibrinogen, collagens IV and VI, to invade host cells and to induce cell death *in vitro*. We analysed the possible association of these results with characteristics such as methicillin resistance, Panton–Valentine leucocidin (PVL) positivity and clonality. The *S. aureus* isolates displayed diversity in their abilities to adhere to various human ligands. All isolates were highly invasive except for ST121. PVL-negative isolates were significantly more invasive than the PVL-positive isolates (p 0.004). Isolates of CC5, CC30 and CC121 were non-cytotoxic, whereas isolates of CC22, CC8, CC15, CC45 and CC88 were very cytotoxic. No statistical association was identified between cell death and methicillin resistance, bacterial PVL status, clonality or patient HIV status. The vast majority of isolates were invasive and induced significant cell death. PVL-negative isolates were more invasive than PVL-positive isolates, while methicillin-resistant isolates were not found to be more invasive or cytotoxic than methicillin-susceptible isolates.

## Introduction

*Staphylococcus aureus* is a facultative intracellular bacterium and a significant human pathogen. It possesses many surface factors that aid with host colonization and cellular invasion as well as secreted virulence factors involved in host cell death induction 1.

Fibronectin, fibrinogen and collagen are three of many extracellular matrix molecules found in macromolecular structures. Both fibronectin and fibrinogen play significant roles in the adherence of *S. aureus* during infections associated with skin diseases such as atopic dermatitis 2. Fibronectin is also a component of human plasma and connective tissue 3. Fibrinogen-binding is commonly associated with infective endocarditis 4, whereas collagen binding is commonly required for the colonization of cartilage 5. Numerous bacterial surface proteins can be used during the process of adherence to host ligands and are called ‘microbial surface components recognizing adhesive matrix molecules’ or MSCRAMM, such as fibronectin-binding proteins A and B 6, staphylococcal protein A and clumping factors. Another group of bacterial proteins which is involved in this process are the SERAM molecules, or the ‘secreted expanded repertoire adhesive molecules’ 7, such as the extracellular adherence protein (Eap). Adherence to fibronectin by *S. aureus* can be mediated by fibronectin-binding proteins A and B (FnbA/B), which also aid in the binding of the organism to plasma clots 8. Both genes are fundamental for the invasion of eukaryotic cells 7. *Staphylococcus aureus* possess two distinct fibrinogen-binding proteins, namely clumping-factor A and B, of which clumping factor A is mainly used to adhere to substances containing fibrinogen 8.

Many groups have clearly demonstrated the role of Fnb proteins as the main invasin of *S. aureus* and identified a fibronectin-dependent bridging mechanism to the host cellular integrin α_5_β_1_ 9. Fibronectin-binding proteins do not require any other *S. aureus*-specific co-receptors to confer invasiveness and this function can be accomplished by either FnbA or FnbB 10, which must be anchored into the bacterial cell wall, as truncation of these proteins results in deficient adherence and cellular invasiveness 11. It has been shown that the extracellular adherence protein, with its broad binding capacity, can play a role in the cellular invasion of host cells 12.

In any given *S. aureus* strain, host cell death induction is difficult to predict and depends on many factors 13. Various bacterial virulence factors are involved, of which α-toxin 14 is described as the most prominent. Intracellular *S. aureus*, if viable, can exist free in the cytoplasm and kill endothelial cells, partly by apoptosis 15. Metabolically active intracellular staphylococci are required for the induction of apoptosis in endothelial cells, which is dependent on *agr* and *sigB* 16. Strains with invasive and haemolytic phenotypes are normally associated with caspase-dependent induction of apoptosis, while non-invasive haemolytic or non-haemolytic invasive isolates are not 16.

Another well-characterized virulence factor of *S. aureus* that can be responsible for host cell death induction, especially of human neutrophils 17, is the two-component leucotoxin, Panton–Valentine leucocidin (PVL). This toxin has been associated with necrotizing pneumonia 18, skin-and-soft tissue infections (SSTI) 19 and necrotizing lesions of the skin and subcutaneous tissues 20 and is very common among diverse genetic backgrounds associated with community-acquired methicillin-resistant *S. aureus* (MRSA), especially the USA300 clone 21.

The aim of this research was to investigate the abilities of *S. aureus* isolates representative of clones causing infection in our patient population to adhere to immobilized ligands, to investigate their cellular invasiveness and host cell death induction abilities, and to identify any associations between adherence, invasiveness or cell death induction and bacterial characteristics, such as methicillin resistance, PVL positivity and clonality.

## Materials and Methods

### Selection of representative isolates (*n* = 25)

From a collection of 367 well-characterized clinical *S. aureus* isolates originating from patients in the Western Cape 22, South Africa, a representative isolate was randomly selected from each major and intermediate pulsed-field gel electrophoresis clone. Two isolates from minor clones statistically associated with HIV infection and two isolates selected from the HIV-positive patients from the dominant MRSA and methicillin-susceptible *S. aureus* (MSSA) clones were also included to investigate any specific associations with HIV infection. An MRSA isolate with a non-typeable SCC*mec* element was also included as representative of a unique local clone.

### Bacterial strains

All bacterial isolates were stored at −80°C until further testing. The following isolates were used as controls: NCTC8325-4 (adherence), Cowan I (invasive control isolate), *Staphylococcus carnosus* TM300 (non-invasive control and non-cytotoxic control isolate) and 6850 (cytotoxic control isolate).

### Adherence assay

Adherence was tested first in uncoated plates to establish a baseline. Then, 96-well plates (Sarstedt, Nümbrecht, Germany) were coated with a specific ligand using a modified method of Peacock *et al*. 23. Standardized bacterial cultures were used at an OD_600_ = 1 in triplicate with three independent experiments. The plate was inoculated with bacterial culture, incubated at 37°C/5% CO_2_ overnight, washed with PBS and stained using 0.1% crystal violet solution. After this, the plate was washed with PBS, eluted with 1% SDS at room temperature overnight, and subsequently measured with an ELISA reader (TECAN Infinite Pro 200, Männedorf, Switzerland) at 620 nm. Adherence to a specific ligand was expressed as a percentage relative to the positive control after subtraction of the PBS negative control. The mean of the means and standard error of mean (SEM) were determined.

### Mammalian cell culture

293 cells (www.atcc.org) were used to investigate the cellular invasiveness. 293 cells were maintained in Dulbecco's modified Eagle's medium (DMEM)/F-12 (Invitrogen, Carlsbad, CA, USA) supplemented with 10% fetal calf serum (PAA Laboratories, Pasching, Austria) and 1× Pen/strep mix (100 U/mL penicillin and 100 μg/mL streptomycin) (Cambrex Bio Science, Verviers, Belgium) and maintained in humidified air (37°C/5% CO_2_). Ea.hy926 cells (www.atcc.org) were used to investigate cell death induction. Ea.hy926 cells were maintained in DMEM/F-12 supplemented with 10% fetal calf serum and 1× penicillin/streptomycin mix (100 U/mL penicillin and 100 μg/mL streptomycin) and maintained in humidified air (37°C/5% CO_2_).

### FACS invasion assay

The cellular invasiveness was determined by adapting a previously published FACS-based invasion assay 10. Briefly, 293 cells were plated in 24-well plates at 3 × 10^5^ cells/well the day before the assay. The day of the assay, the cells were washed once with 500 μL invasion medium (1% human serum albumin and 10 mM HEPES in DMEM), 500 μL invasion medium was added to each well and the plate was pre-cooled at 4°C for 20 min. Following this, 50 μL bacterial suspension (adjusted to an OD_540_ = 1) was added to each well (MOI ∼30) and incubated for 1 h at 4°C. Thereafter, the cells were shifted to 37°C with 5% CO_2_ for 3 h to allow for bacterial invasion. The cells were harvested and analysed by FACS analyses as previously described 9.

All bacterial suspensions were adjusted to an OD_540_ = 1. Results obtained were expressed as the average of the mean values with the SEM. An arbitrary cut-off value of 50% was used to discriminate between invasive (≥50%) and non-invasive (<50%) isolates.

### Cell death/viability assays

Bacteria were grown as described previously and standardized to an OD_540_ = 1. Ea.hy926 cells were plated in 24-well plates at 3 × 10^5^ cells/well for the Nicoletti assay or 96-well plates at 5 × 10^4^ cells/well for the lactate dehydrogenase (LDH) and WST-1 assays and infected at an MOI ∼30. Results obtained were expressed as the average of the mean values with the SEM. An arbitrary cut-off value of 50% was used to discriminate between cytotoxic (≥50%) and non-/moderately cytotoxic (<50%) isolates.

### Nicoletti assay

An adapted version of this previously published protocol was used 16. The percentage of intact cells, relative to the cells-only control (set at 100%), was determined and the percentage of dead cells was calculated.

### LDH assay

This assay was performed using the LDH cytotoxicity kit (Roche Diagnostics, Mannheim, Germany) as per the manufacturers' recommendation. The absorbance was measured using a Tecan Reader Infinite F200 Pro (Tecan GmbH, Crailsheim, Germany) at 490 nm, with a reference reading at >600 nm. Cell death induction was expressed as a percentage relative to the 1% Triton-X control (set at 100%).

### WST-1 cell viability assay

The WST-1 ready-to-use reagent (Roche Diagnostics) was used as per the manufacturers' recommendations. The absorbance was measured using a Tecan Reader Infinite 200 Pro (Tecan Group, Männedorf, Switzerland) at 490 nm, with a reference reading at >600 nm. Cell viability was expressed as a percentage relative to the cells-only control (set at 100%). Results obtained were expressed as % cell death induction for direct comparison with the Nicoletti and LDH assays.

### Statistical investigations

#### Associations between adherence, invasion or cell death induction and methicillin-resistance, clonality, patient HIV status or bacterial PVL status

For associations between adherence, invasion or cell death induction and methicillin-resistance, two tests were performed: the two-sample *t*-test and the two-sample Wilcoxon rank-sum test (Mann–Whitney test). The Mann–Whitney test was also used to investigate associations between adherence, invasion or cell death induction and bacterial PVL status. For associations between adherence, invasion or cell death induction and clonality/patient HIV status, the Kruskal–Wallis test was performed. The singletons were grouped together. For all statistical investigations, a confidence interval of 95% was used and an association was regarded as statistically significant if a p <0.05 was obtained. All statistical analyses were performed with the software package Stat v.12.

#### Correlation of *in vitro* data

We investigated correlations between the *in vitro* data generated by calculating the correlation coefficient (*r*) and the statistical significance thereof (p value). The software package Statistica v.10 was used.

## Results

### Adherence assays

**Figure 1:**
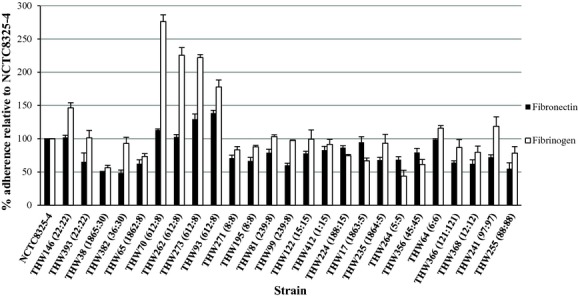
*In vitro* adherence of the representative isolates to fibronectin (black bars) and fibrinogen (white bars). The number next to each isolate's name is the MLST ST:MLST CC. The data displayed are representative of three independent experiments performed in triplicate and relative to NCTC8325-4 as the control. MLST, Multi locus sequence typing.

**Figure 2:**
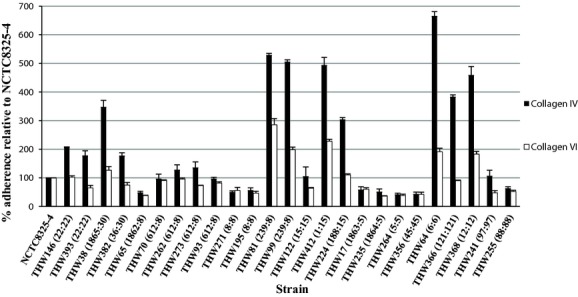
*In vitro* adherence of the representative isolates to collagen IV (black bars) and collagen VI (white bars). The number next to each isolate's name is the MLST ST:MLST CC. The data displayed are representative of three independent experiments performed in triplicate and relative to NCTC8325-4 as the control. MLST, Multi locus sequence typing.

The selected isolates displayed variability in their adherence to the different immobilized ligands. Some isolates displayed a preference for adherence to fibronectin and fibrinogen (CC8 and CC5) (Fig.1) whereas others (CC30, CC15 and ST239) displayed preferences for adherence to collagen IV and VI (Fig.2). CC22 adhered strongly to immobilized fibrinogen, fibronectin, collagen IV and VI. Some isolates such as ST88, ST97 and ST45 isolates were poor binders (Figs.1 and 2). MRSA isolates displayed stronger adherence to plasma (p 0.025) (Table1). PVL-negative isolates adhered more strongly in the absence of a ligand (p 0.01) and fibronectin (p 0.034) compared to PVL-positive isolates (Table1).

**Table 1:** Statistical associations between adherence, invasion and cell death and methicillin-resistance, bacterial Panton–Valentine leucocidin status, clonality or patient HIV status

Ligand	p value—MRSA/MSSA^a^	p value—MRSA/MSSA^b^	p value—PVL^a^	p value—clonality^c^	p value—HIV^c^
**Uncoated**	0.767	**0.016 (MRSA)**	**0.010 (PVL**−**)**	0.194	0.322
Serum	0.613	0.365	0.364	0.152	0.506
**Plasma**	0.767	**0.025 (MRSA)**	0.250	0.530	0.849
**Fibronectin**	0.567	0.335	**0.034 (PVL**−**)**	0.201	0.581
Fibrinogen	0.680	0.080	0.468	0.160	0.210
Collagen IV	0.440	0.505	0.364	0.248	0.104
Collagen VI	0.553	0.758	0.904	0.292	0.055
**Invasion**	0.681	0.112	**0.004 (PVL**−**)**	**0.004 (CC8)**	0.628
Nicoletti	0.680	0.901	0.758	0.758	0.089
LDH	0.507	0.910	0.586	0.586	0.829
WST-1	0.577	0.319	0.525	0.525	0.101

Statistically significant values are displayed in bold (p <0.05). LDH, lactate dehydrogenase; MRSA, methicillin-resistant *Staphylococcus aureus*; MSSA, methicillin-susceptible *Staphylococcus aureus*; PVL, Panton-Valentine leukocidin.

a Mann–Whitney U-test.

b t-test.

c Kruskal–Wallis test.

### Cellular invasiveness

**Figure 3:**
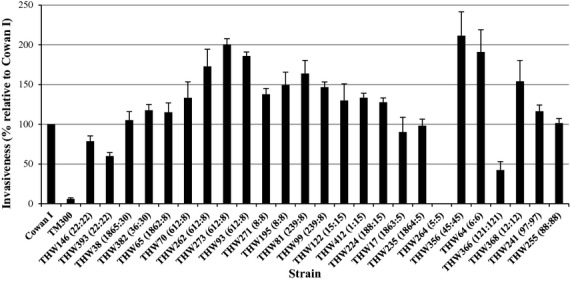
Invasiveness of representative South African *Staphylococcus aureus* isolates determined by FACS-based invasion assay on 293 cells. The number next to each isolate's name is the MLST ST:MLST CC. Invasiveness is expressed as a percentage relative to Cowan I for three independent experiments performed in duplicate. MLST, Multi locus sequence typing.

The cellular invasiveness of the isolates was diverse (42.4–211.6%; mean 142.6%; median 142.2%) (Fig.3). All isolates, irrespective of the multilocus sequence typing clonal complex (MLST CC), were invasive except for THW-366 (ST121, MSSA PVL+). Two isolates were classified as highly invasive (>200%): THW-273 (ST612:CC8; SCC*mec* IV; PVL− MRSA) and THW-356 (ST45:CC45; MSSA PVL−). We were unable to determine the invasiveness of isolate THW-264 (ST5:CC5; SCC*mec* I; PVL− MRSA). No significant difference in invasiveness was observed between isolates from HIV-positive or HIV-negative patients.

### Cell death assays

#### Nicoletti assay

**Figure 4:**
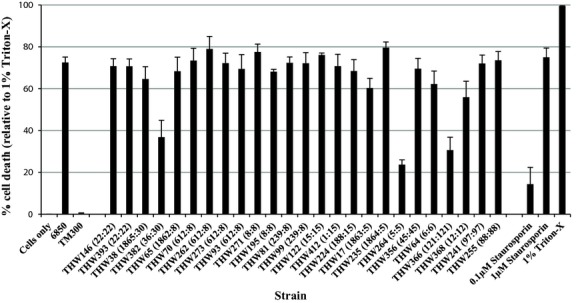
Induction of host cell death of representative South African *Staphylococcus aureus* isolates determined by the Nicoletti assay on EA.hy926 cells. The number next to each isolate's name is the MLST ST:MLST CC. Cytotoxicity is expressed as a percentage relative to the cells only control of three independent experiments performed in duplicate. MLST, Multi locus sequence typing.

Host cell death induction was diverse (23.7–79.6%; mean = 65.4%; median = 69.6%), although 21 of the isolates killed >60% of cells (Fig.4). The following isolates were classified as non-cytotoxic: THW-382 (ST36:CC30; SCC*mec* II; PVL-negative MRSA); THW-264 (ST5:CC5; SCC*mec* I; PVL-negative MRSA) and THW-366 (ST121:CC121; PVL-positive MSSA; SSTI). Thirteen isolates could be classified as very cytotoxic (>70% cell death) (CC22, CC8, CC15, CC5, CC97, CC88).

#### LDH assay

Host cell death induction was diverse (34.1–94.3%; mean = 61.4%; median = 59.6%) (see Supporting information, Fig. S1). Twenty-two isolates were cytotoxic and three were non-cytotoxic, of which two were also classified as non-cytotoxic using the Nicoletti assay (THW264; THW-366). THW-99 (ST239:CC8; SCC*mec* NT) was also non-cytotoxic using this assay. Nine isolates could be classified as very cytotoxic (>70% cell death) (CC22, CC30, CC8, CC15, CC45, CC88).

#### WST-1 assay

Host cell death induction was diverse (68–27.1%; mean = 38.1%; median = 36%) (data not shown). Twenty-two isolates were cytotoxic and three were non-cytotoxic, of which the same two isolates were previously classified as non-cytotoxic using the Nicoletti and LDH assays. The following isolate was also non-cytotoxic using this assay: (1) THW-81 (ST239:CC8; PVL-negative MRSA). Using this assay, seven isolates could be characterized as very cytotoxic (>70% viable cells) (CC22, CC8, CC45, CC88).

Two isolates were classified as non-cytotoxic using all three assays: (1) THW-366 (ST121:CC121; PVL-positive MSSA; SSTI); (2) THW-264 (ST5:CC5; SCC*mec* I; PVL-negative MRSA).

### Statistical associations

MRSA isolates adhered more strongly to plasma proteins than MSSA isolates, whereas PVL-negative isolates adhered more strongly to fibronectin compared with PVL-positive isolates (Table1). CC8 isolates were the only isolates statistically associated with being invasive. PVL-negative isolates were significantly more invasive than the PVL-positive isolates (Table1). Regarding correlation analyses, the Pearson correlation coefficient (*r*) and respective p-values for correlations between the *in vitro* assays can be found in the Supporting information, Table S2.

## Discussion

Our collection of isolates displayed a diverse range of adherence to the different ligands. ST612 was the dominant MRSA clone identified and has previously been reported in Australia 24 and South Africa 25. Fibronectin and fibrinogen may play a role in SSTI. Two of the ST612 isolates were isolated from SSTI. Elgalai *et al*. also described a collection of wound isolates that displayed a diverse range of adherence and similarly to our set of isolates, displayed stronger adherence to fibrinogen than to fibronectin 26. Seidl *et al*. described no significant adherence potentials to immobilized fibronectin between isolates derived from persistent and resolving MRSA infections 27. Unlike previously reported by Arciola *et al*. 28, adherence to both human collagen IV and VI varied greatly among the isolates. The reason for the stronger adherence seen by MRSA isolates to human serum proteins, as well as for PVL-negative isolates to fibronectin, remains unclear, as all isolates tested positive for *fnbA* and *fnbB* (data not shown). We might speculate that the clonal lineages associated with methicillin-resistance and PVL-negativity prefer a preference for adherence to these ligands.

We were able to establish that 23/25 isolates were invasive. No association could be identified between invasion and methicillin-resistance. An association between clonality and invasiveness was only identified for CC8 isolates, the only CC with a large enough number of isolates to allow for this analysis. All CC8 isolates were classified as being invasive and displayed a high degree of cellular invasiveness (range 133.3–200.5%). Fowler *et al*. also previously reported that most *S. aureus* genotypes are capable of causing invasive diseases, but that CC5 and CC30 isolates displayed greater levels of haematogenous complication 29. Zautner *et al*. identified a variety of CCs associated with recurrent tonsillitis-causing isolates as a result of intracellular persisting *S. aureus*. They characterized these isolates and identified many of them to be invasive isolates from CC30, CC45, CC8, CC5, CC15 and CC22 30. Park *et al*. investigated the association between the invasiveness of clinical *S. aureus* isolates and their abilities to cause metastatic infections 31. They found that invasiveness of endothelial cells is not a major determinant of metastatic infections. When investigating the expression patterns of human umbilical endothelial cells upon internalization of *S. aureus*, Stark *et al*. found that innate immune responses dominated, irrespective of the invasiveness of the bacterial isolates 32. THW-366 (ST121; PVL+ MSSA; associated with SSTI; 42.4% invasiveness) was the only isolate classified as non-invasive and also the only *agr* IV isolate. This ST has previously been associated with community acquired MRSA SSTI in children in Portugal 33. No data on the invasiveness were available from these studies. Our data contradict data published by Zautner *et al*., who identified three ST121 isolates associated with recurrent tonsillitis. The isolates displayed invasiveness of 33.7%, 56.5% and 65.3% relative to Cowan I, the invasive control 30. THW-264 (ST5:CC5; SCC*mec* I; PVL− MRSA) is another isolate of interest as we were unable to determine the invasiveness of this isolate due to technical difficulties. We speculate that this isolate might be *pls*^*+*^, since this gene is commonly associated with SCC*mec* I. It has previously been demonstrated that MRSA isolates expressing *pls* displayed a significantly reduced ability to invade host cells 34. We hypothesize that this isolate might be non-invasive. This isolate was collected from a paediatric patient suffering from conjunctivitis.

Twenty-three isolates were cytotoxic (>50% cell death using all three assays) and the remaining two isolates were non-cytotoxic (<50% cell death using all three assays). No association was identified between cell death and methicillin-resistance or clonality. Four isolates stood out as they were the only isolates classified as very cytotoxic according to all three assays, resulting in >70% cell death for every assay (THW-262 (ST612-MRSA-IV), THW-70 (ST612-MRSA-IV); THW-356 (ST45-MSSA) and THW-255 (ST88-MRSA-IV)). These data show that PVL− isolates (THW262; THW70; THW271; THW273; THW412; THW356) can be as cytotoxic as PVL+ isolates (THW146; THW393; THW255) and a very high degree of cytotoxicity was even more common in PVL− isolates. A possible cause of this phenotypic display of high levels of cytotoxicity might be due to the action of phenol soluble modulins, as it has been shown that phenol soluble modulin-*α* is required for phagosomal escape following phagocytosis by non-professional phagocytes, allowing for intracellular bacterial replication and dissemination 22.

The same applied for MSSA and MRSA isolates. Highly cytotoxic MRSA and MSSA isolates were identified. Three highly cytotoxic MRSA isolates were identified (THW262; THW70; THW255) and one highly cytotoxic MSSA isolate was identified (THW356). Also, one non-cytotoxic MSSA and MRSA isolate was identified. It is interesting to note that the most cytotoxic MRSA isolates all carry SCC*mec* IV. This is of interest because ST88 is associated with community-acquired MRSA. ST612 has previously been described from hospital isolates in South Africa. Four isolates were classified as very cytotoxic according to the Nicoletti and LDH assays only. We can speculate that these isolates are capable of inducing host cell death using both apoptotic and necrotic mechanisms. These isolates were identified from a pus swab of the tibia of an HIV-positive man, fluid from a sinus fungal infection and pus from a hand infection from another HIV-positive female. Park *et al*. investigated if any association was present between cytotoxicity of clinical *S. aureus* isolates and their abilities to cause metastatic infections 31. They found that the ability to induce host cell death and be cytotoxic to endothelial cells was not a major determinant of metastatic infections. During the investigation of ten MRSA (CC45 and CC5) isolates collected during a national clinical trial, Seidl *et al*. determined that all ten isolates were invasive in human endothelial cells, but only some of the isolates were cytotoxic 13. Our data contradict those of Krut *et al*., who published cytotoxicity data for a collection of *S. aureus* isolates, which displayed cytotoxicity to be a strain-specific characteristic, and more than half of the collection of isolates were classified as non-cytotoxic 35. It is worth noting that the study conducted by Krut *et al*. focused on murine cell lines, whereas our study used a human cell line to investigate host cell death induction.

## Conclusion

Clinical *S. aureus* isolates collected at Tygerberg hospital displayed diversity in their abilities to adhere to various immobilized human ligands. MRSA isolates displayed stronger adherence to human plasma proteins than MSSA isolates, while PVL− isolates adhered more strongly to fibronectin and were as cytotoxic as PVL+ isolates. The vast majority of clinical *S. aureus* isolates included in this study were invasive, although differences in the degree of invasiveness were common between different CCs. ST121 was the only ST in this collection classified as non-invasive. The vast majority of these isolates are also able to induce the death of host cells. No differences in cellular invasiveness or cytotoxicity of isolates from HIV-positive and/or HIV-negative persons were detectable. Isolates containing virulence factors such as PVL or methicillin-resistant isolates were not found to be more invasive or cytotoxic than PVL− or MSSA isolates.

### Study limitation

Isolates used were a convenience sample and bias could have been introduced through the selection. The statistical analyses performed assume that a representative random sample of isolates were constituted.

## Supporting Information

**Table S1.** Strains selected as representative isolates **Table S2.** Pearson correlation (*r*) for the correlation between different assays and the corresponding p-value. Statistically significant (p <0.05) correlations are displayed in bold **Fig. S1.** Induction of host cell death of representative South African *Staphylococcus aureus* isolates determined by lactate dehydrogenase (LDH) assay on EA.hy926 cells. The number next to each isolates name is the MLST ST:MLST CC. Cytotoxicity is expressed as a percentage relative to the Triton-X control of three independent experiments performed in triplicate.
