# Supplementary material for: In vitro characterization of representative clinical South African Staphylococcus aureus isolates from various clonal lineages
Source: New Microbes New Infect. 2014 Jun 26;2(4):115–22. doi: 10.1002/nmi2.53 (PMC4184580; doi:10.1002/nmi2.53)
Supplement: Supplementary file 1 — Table S1. Strains selected as representative isolates Table S2. Pearson correlation (r) for the correlation between different assays and the corresponding p-value. Statistically significant (p <0.05) correlations are displayed in bold Fig. S1. Induction of host cell death of representative South African Staphylococcus aureus isolates determined by lactate dehydrogenase (LDH) assay on EA.hy926 cells. The number next to each isolates name is the MLST ST:MLST CC. Cytotoxicity is expressed as a percentage relative to the Triton-X control of three independent experiments performed in triplicate. [file nmi20002-0115-sd1.docx]

**SUPPLEMENTARY TABLES:**

##### Table S1 Strains selected as representative isolates.

| **Isolate**  **name** | **PFGE** | **Clinical data** | **Source** | **Status of PFGE clone** | **MLST**  **ST** | **MLST**  **CC** | ***spa*-CC** | **SCC*mec*** | ***agr*** | **PVL** | **Statistical association** |
| --- | --- | --- | --- | --- | --- | --- | --- | --- | --- | --- | --- |
| THW38 | THW-A | Lip abscess | SSTI | Major | 1865 | 30 | 21 | NA | III | + | MSSA/PVL+/SSTI |
| THW382 | THW-B | Laparotomy  (day 15 post-operative) | SSTI | Inter | 36 | 30 | 21 | II | III | - | MRSA/PVL- |
| THW146 | THW-C | Abscess left knee | SSTI | Major | 22 | 22 | 891 | NA | I | + | MSSA/PVL+/SSTI |
| THW393 | THW-C | Septic left tibia ex-fix | BJ | Major | 22 | 22 | 891 | NA | I | + | None^1^ |
| THW366 | THW-E | Chronic folliculitis | SSTI | Major | 121 | 121 | NF:16 | NA | IV | + | MSSA/PVL+/SSTI |
| THW262 | THW-O | Pyrexia, intracostal drainage site | RT | Major | 612 | 8 | 64 | IV | I | - | MRSA/PVL- |
| THW65 | THW-G | Septic ORIF | BJ | Inter | 1862 | 8 | 1597 | NA | I | - | MSSA |
| THW271 | THW-J | Sinus fungal infection | ENT | Inter | 8 | 8 | 64 | V | I | - | PVL- |
| THW81 | THW-L | Sepsis, pin tract | PROST | Inter | 239 | 8 | 21 | III | I | - | MRSA/PVL- |
| THW70 | THW-N | Sepsis, nosocomial MRSA | SSTI | Inter | 612 | 8 | 64 | IV | I | - | MRSA/PVL- |
| THW195 | THW-K | Impetigo | SSTI | Minor | 8 | 8 | 64 | NA | I | - | HIV+ |
| THW273 | THW-P | Pus from hand | SSTI | Minor | 612 | 8 | 64 | IV | I | - | HIV+ |
| THW93 | THW-O | Sepsis | UK | Major | 612 | 8 | 64 | IV | I | - | None^1^ |
| THW99 | THW-L | Septic wound | SSTI | Inter | 239 | 8 | 21 | UK | I | - | None^2^ |
| THW122 | THW-S | Sepsis | SSTI | Major | 15 | 15 | 84 | NA | II | - | MSSA/PVL- |
| THW412 | THW-V2^@^ | No clinical data supplied | UT | Major | 1 | 15 | NF:14 | NA | III | - | MSSA |
| THW224 | THX-X | Wound sepsis | SSTI | Inter | 188 | 15 | EX | NA | I | - | MSSA/PVL- |
| THW17 | THW-Y | Wound sepsis | SSTI | Major | 1863 | 5 | 2 | NA | II | - | MSSA/PVL- |
| THW235 | THW-Z | Measles | EY | Inter | 1864 | 5 | 2 | NA | II | + | MSSA |
| THW264 | THW-AA | Conjunctivitis | EY | Inter | 5 | 5 | 2 | I | II | - | MRSA/PVL- |
| THW64 | THW-V1^@^ | Suspect TB, pericardial fluid | CA | Major | 6 | 6 | 64 | NA | I | - | MSSA/PVL- |
| THW356 | THW-EE | Hip abscess | SSTI | Major | 45 | 45 | EX | NA | I | - | MSSA/PVL- |
| THW368 | THW-V3^@^ | Wound infection | SSTI | Inter | 12 | 12 | 888 | NA | II | + | MSSA/PVL+ |
| THW241 | THW-W | ESRF | IVD | Inter | 97 | 97 | 267 | NA | I | - | MSSA/PVL- |
| THW255 | THW-BB | Infected leg | SSTI | Inter | 88 | 88 | 186 | IV | III | + | PVL+ |

ESFR = end-stage renal failure; ORIF = open reduction and internal fixation; Inter = intermediate; NA = not applicable; Ex = excluded; SSTI = skin and soft tissue; BJ = bone and joint; RT = respiratory tract; ENT = ear, nose and throat; PROST = prosthetic device; UK = unknown; UT = urinary tract; EY = eye; CA = cardiac; IVD = intravascular device; 1 = selected from HIV+ patients of dominant MRSA and MSSA clones; 2 = only MRSA isolates identified with a non-typeable SCC*mec* element; @ = *Apa*I MRA

**Table S2** Pearson correlation (r) for the correlation between different assays and the corresponding p-value. Statistically significant (p < 0.05) correlations are displayed in bold.

| **Assay** | **Pearson correlation (r)** | **p-value** | **Figure** |
| --- | --- | --- | --- |
| **Correlations between adherence assays** | | | |
| Uncoated vs. serum | 0.0498 | 0.830 | S3 |
| **Uncoated vs. plasma** | **0.7046** | **0.00008** | **S4** |
| **Uncoated vs. fibronectin** | **0.6025** | **0.0014** | **S5** |
| **Uncoated vs. fibrinogen** | **0.6277** | **0.0008** | **S6** |
| Uncoated vs. collagen IV | -0.3363 | 0.102 | S7 |
| Uncoated vs. collagen VI | -0.2131 | 0.3065 | S8 |
| Plasma vs. serum | 0.3256 | 0.1123 | S10 |
| **Plasma vs. fibronectin** | **0.7099** | **0.00007** | **S11** |
| **Plasma vs. fibrinogen** | **0.7842** | **0.000001** | **S12** |
| Plasma vs. collagen IV | -0.0596 | 0.7771 | S13 |
| Plasma vs. collagen VI | 0.0122 | 0.9538 | S14 |
| **Serum vs. fibronectin** | **0.4997** | **0.0110** | **S15** |
| Serum vs. fibrinogen | 0.3481 | 0.0882 | S16 |
| Serum vs. collagen IV | 0.0791 | 0.7069 | S17 |
| Serum vs. collagen VI | 0.1674 | 0.4238 | S18 |
| **Fibronectin vs. fibrinogen** | **0.7439** | **0.00002** | **S19** |
| Fibronectin vs. collagen IV | -0.0852 | 0.6854 | S20 |
| Fibronectin vs. collagen VI | 0.0339 | 0.8721 | S21 |
| Fibrinogen vs. collagen IV | -0.1055 | 0.6157 | S22 |
| Fibrinogen vs. collagen VI | 0.0195 | 0.9262 | S23 |
| **Collagen IV vs. collagen VI** | **0.9048** | **0.00001** | **S24** |
| **Correlations between adherence and invasion assays** | | | |
| Uncoated vs. invasion | 0.3279 | 0.1179 | S25 |
| **Serum vs. invasion** | **0.5397** | **0.0065** | **S26** |
| Plasma vs. invasion | 0.3923 | 0.0579 | S27 |
| **Fibronectin vs. invasion** | **0.4702** | **0.0204** | **S28** |
| Fibrinogen vs. invasion | 0.3121 | 0.1376 | S29 |
| Collagen IV vs. invasion | 0.1054 | 0.6241 | S30 |
| Collagen VI vs. invasion | 0.2418 | 0.2549 | S31 |
| **Correlations between adherence and cell death assays** | | | |
| Serum vs. Nicoletti | 0.2947 | 0.1527 | S32 |
| Uncoated vs. Nicoletti | -0.0577 | 0.7840 | S33 |
| Plasma vs. Nicoletti | 0.0449 | 0.8313 | S34 |
| Fibronectin vs. Nicoletti | 0.2985 | 0.1473 | S35 |
| Fibrinogen vs. Nicoletti | 0.3569 | 0.0799 | S36 |
| Collagen IV vs. Nicoletti | -0.1097 | 0.6017 | S37 |
| Collagen VI vs. Nicoletti | 0.0741 | 0.7249 | S38 |
| Serum vs. LDH | 0.1975 | 0.3440 | S39 |
| Uncoated vs. LDH | 0.0157 | 0.9408 | S40 |
| Plasma vs. LDH | 0.1604 | 0.4436 | S41 |
| Fibronectin vs. LDH | 0.0799 | 0.7043 | S42 |
| Fibrinogen vs. LDH | 0.3179 | 0.1215 | S43 |
| Collagen IV vs. LDH | -0.1981 | 0.3424 | S44 |
| Collagen VI vs. LDH | -0.0911 | 0.6651 | S45 |
| Serum vs. WST-1 | -0.1107 | 0.5985 | S46 |
| Uncoated vs. WST-1 | 0.0078 | 0.9705 | S47 |
| Plasma vs. WST-1 | -0.0976 | 0.6426 | S48 |
| Fibronectin vs. WST-1 | -0.2138 | 0.3049 | S49 |
| Fibrinogen vs. WST-1 | -0.3662 | 0.0718 | S50 |
| Collagen IV vs. WST-1 | 0.3668 | 0.0713 | S51 |
| Collagen VI vs. WST-1 | 0.3006 | 0.1442 | S52 |
| **Correlations between invasion and cell death assays** | | | |
| Invasion vs. Nicoletti | 0.3283 | 0.1173 | S53 |
| Invasion vs. LDH | 0.0768 | 0.7213 | S54 |
| Invasion vs. WST-1 | 0.0033 | 0.9880 | S55 |
| **Correlations between difference cell death assays** | | | |
| **Nicoletti vs. LDH** | **0.5459** | **0.0048** | **S56** |
| **Nicoletti vs. WST-1** | **-0.7405** | **0.00002** | **S57** |
| **WST-1 vs. LDH** | **-0.6774** | **0.0002** | **S58** |

**SUPPLEMENTARY FIGURES:**

**Figure S1** Induction of host cell death of representative South African *S. aureus* isolates determined by LDH assay on EA.hy926 cells. The number next to each isolates names is the MLST ST:MLST CC. Cytotoxicity is expressed as a percentage relative to the Triton-X control of three independent experiments performed in triplicate.
